# Transcriptional Profiling of Porcine Blastocysts Produced In Vitro in a Chemically Defined Culture Medium

**DOI:** 10.3390/ani11051414

**Published:** 2021-05-14

**Authors:** Josep M. Cambra, Emilio A. Martinez, Heriberto Rodriguez-Martinez, Maria A. Gil, Cristina Cuello

**Affiliations:** 1 Department of Medicine and Animal Surgery, Faculty of Veterinary Medicine, International Excellence Campus for Higher Education and Research, University of Murcia, 30100 Murcia, Spain; josepmiquel.cambra@um.es (J.M.C.); emilio@um.es (E.A.M.); ccuello@um.es (C.C.); 2 Institute for Biomedical Research of Murcia (IMIB-Arrixaca), Campus de Ciencias de la Salud, Carretera Buenavista s/n, El Palmar, 30120 Murcia, Spain; 3 Department of Biomedical & Clinical Sciences (BKV), BKH/Obstetrics & Gynecology, Faculty of Medicine and Health Sciences, Linköping University, SE-58185 Linköping, Sweden; heriberto.rodriguez-martinez@liu.se

**Keywords:** in vitro, embryo, porcine, defined media, PF4, BSA, transcriptome, microarray

## Abstract

**Simple Summary:**

The development of chemically defined media has become a particularly important task for in vitro embryo production systems, which require maintained reproducible results when new additives are tested for culture, beyond observational studies. Specifically, we need studies measuring the impact of these media on the embryonic transcriptome, particularly those negatively affecting embryo quality. Consequently, this study evaluated by using a microarray approach the transcriptome of porcine embryos produced in vitro, cultured in a defined vs. an undefined medium and contrasted with in vivo-derived embryos. No significantly altered genes were found between in vitro-produced embryos, despite the theoretical limitations that usually accompany defined media. However, when they were compared with in vivo-derived embryos, many altered genes were observed, reflecting how current culture conditions deeply modify the embryonic transcriptome. A better understanding of these alterations may offer new ways to improve in vitro embryo production systems. Likewise, developing a chemically defined medium capable of producing embryos of a similar quality to traditional media may contribute to this task.

**Abstract:**

The development of chemically defined media is a growing trend in in vitro embryo production (IVP). Recently, traditional undefined culture medium with bovine serum albumin (BSA) has been successfully replaced by a chemically defined medium using substances with embryotrophic properties such as platelet factor 4 (PF4). Although the use of this medium sustains IVP, the impact of defined media on the embryonic transcriptome has not been fully elucidated. This study analyzed the transcriptome of porcine IVP blastocysts, cultured in defined (PF4 group) and undefined media (BSA group) by microarrays. In vivo-derived blastocysts (IVV group) were used as a standard of maximum embryo quality. The results showed no differentially expressed genes (DEG) between the PF4 and BSA groups. However, a total of 2780 and 2577 DEGs were detected when comparing the PF4 or the BSA group with the IVV group, respectively. Most of these genes were common in both in vitro groups (2132) and present in some enriched pathways, such as cell cycle, lysosome and/or metabolic pathways. These results show that IVP conditions strongly affect embryo transcriptome and that the defined culture medium with PF4 is a guaranteed replacement for traditional culture with BSA.

## 1. Introduction

The ultimate goal of in vitro embryo production (IVP) is to obtain viable and healthy offspring. However, in the case of swine, piglet production from in vitro-derived embryos is quite limited due to the poor quality of the embryos produced. Current IVP-systems have not been able to match the quality of embryos developed in a physiological environment, which is especially reflected in both transcriptome [1,2,3] and epigenome [4] differences between in vivo and IVP-embryos. These differences lead irremediably to the low success rate associated with the transfer of IVP embryos in swine, with maximum development rates of only 15–20% [5,6,7]. Although there are many factors that influence the poor quality of embryos produced in vitro, inadequate in vitro culture (IVC) conditions are possibly one of the most important. It is well known that IVC of in vivo-derived zygotes leads to the production of poor-quality blastocysts [3,8]. For this reason, improving current culture systems is primary to equalize the quality of the IVP-embryos with their in vivo-derived counterparts. Many attempts have been made to improve culture media to mimic the female reproductive tract environment by using chemically undefined compounds, such as fetal bovine serum, bovine serum albumin (BSA) or even culture systems including feeder layers of cell lines or their conditioned media [9,10,11]. Although these types of compounds/media contain molecules favorable for embryo development such as growth factors, proteins, enzymes, hormones, etc. [12,13,14], their use is controversial. The reproducibility of the results (batch-to-batch variation), the potential risk for pathogen transmission (compounds of animal origin) and possible interactions with potential new additives are the most important problems considered [15].

Therefore, the development of chemically defined media is a growing trend within good cell culture practice [16], making these media particularly suitable for the evaluation of different components during embryogenesis [17]. Despite these benefits, it has been particularly difficult to find defined culture media that can offer the same production rates as undefined media. In the case of porcine embryo culture, several animal protein-free defined media have been developed in the last decade [18,19]. More recently, we also designed a defined medium consisting of North Carolina State University (NCSU)-23 medium supplemented with polyvinyl alcohol (PVA) and the cytokine platelet factor 4 (PF4) [20], a cytokine that modulates the expression of cell cycle genes and with positive effects on growth and differentiation in several stem cell lines. Although these defined media are able to maintain embryo production rates at a similar level to their undefined counterparts, their impact on the total embryonic transcriptome remains unknown.

This study aimed to analyze the transcriptome of in vitro-produced porcine blastocysts cultured in NCSU-23-PVA-PF4-defined medium. These results were compared with the transcriptome of blastocysts also produced in vitro but cultured in our routine undefined medium consisting of NCSU-23 supplemented with BSA. Based in our previous IVC results [20], our starting hypothesis is that the impact of both culture media on the blastocyst transcriptome will be similar. The results were contrasted with in vivo-derived blastocysts, which allowed a better analysis of the differences between embryos produced in vitro, ensuring a greater understanding of the impact that in vitro embryo production can exert on gene expression.

## 2. Materials and Methods

Unless otherwise specified, all chemicals and reagents used during this experiment were supplied by Sigma-Aldrich Co. (Alcobendas, Madrid, Spain).

### 2.1. In Vivo Embryo Production

To obtain the in vivo-derived blastocysts, a total of 10 multiparous hybrid sows (Landrace × Large-White) were selected after weaning. On the following days, sows were examined for signs of estrus twice daily by a skilled operator, allowing snout-to-snout contact with vasectomized boars. Sows were artificially inseminated (AI) with the post-cervical technique at 0 and 24 h using refrigerated semen doses of 40 mL with a concentration of 35 × 106 spermatozoa/mL extended in Beltsville thawing solution [21]. Embryos were surgically collected six days after the first AI as previously described [22]. Briefly, sows were sedated by intramuscular injection of azaperone (2 mg/kg body weight) followed by anesthesia induced by intravenous administration of sodium thiopental (7 mg/kg body weight) and maintained by inhalation of isoflurane (3–5%). The genital tract of the sows was exposed via mid-ventral laparotomy, and the number of corpora lutea was recorded in each ovary. Blastocysts were collected by flushing the proximal portions of the uterine horns with 30 mL of TL-HEPES-PVA [23] and evaluated in situ with a stereomicroscope following the morphological criteria established by the International Embryo Transfer Society [24]. Embryos classified as expanded blastocysts with good or excellent morphology were selected for the experiment.

### 2.2. In Vitro Embryo Production

Embryos were produced in vitro following the protocol used in our laboratory [20]. Briefly, cumulus-oocyte complexes (COCs) were obtained by incising and washing medium-sized follicles (3–6 mm in diameter) from ovaries of prepubertal sows slaughtered at a local abattoir. COCs with homogeneous, dark and granulated cytoplasm and at least two intact layers of cumulus cells were washed in pre-equilibrated defined maturation medium, consisting of TCM-199 (Gibco Life Technologies S.A., Barcelona, Spain) supplemented with 0.55 mM glucose, 0.9 mM sodium pyruvate, 75 mg/mL penicillin, 50 mg/mL streptomycin, 0.1 mg/mL PVA, 0.57 mM cysteine and 10 ng/mL epidermal growth factor. After washing, COCs were incubated in maturation medium supplemented with 10 IU eCG (Folligon, Intervet International B.V., Boxxmeer, The Netherlands) and 10 IU hCG (VeterinCorion, Divasa Farmavic, S.A., Barcelona, Spain) for 22 h in a humidified atmosphere with 5% CO_2_ and at 38 °C and then for an additional 22 h in fresh maturation medium in the absence of hormones.

Once maturation was completed, the COCs were vortexed at 1660 rounds/min for 2 min in TL-HEPES-PVA supplemented with 0.1 mg/mL hyaluronidase. The resulting denuded oocytes were washed in maturation medium and then in fertilization medium, which consisted of Tris-buffered medium [25] supplemented with 2 mM caffeine and 0.2 mg/mL BSA (heat shock fraction, pH 5.2, ≥96%, A8022). The oocytes were transferred to droplets of fertilization medium (40 oocytes per drop) and cocultured with frozen–thawed spermatozoa (3000 spermatozoa per oocyte) for five hours. After the coculture period, the putative zygotes were washed and cultured in NCSU-23 [26] supplemented with 0.3 mM sodium pyruvate and 4.5 mM lactate for two days and in NCSU-23 supplemented with 5.5 mM glucose for an additional four days. In each replicate, a representative group of presumptive zygotes was fixed and stained 18 h post-fertilization to calculate the efficiency of the fertilization (zygotes with two pronuclei from the total oocytes inseminated). To ensure the validity of the replicates, different embryo development parameters were evaluated. On day two, the cleavage rate defined as the number of embryos with two or four cells related to the total number of inseminated oocytes, was calculated. At days five and six, the blastocyst rates for each day were calculated as the number of blastocysts out to the number of cleaved embryos at day two. Finally, at day six, total embryo production was also calculated, defined as the number of blastocysts on day six related to the total number of inseminated oocytes. A parametric distribution of the samples was determined using the Shapiro–Wilk test, with means compared using Student’s *t*-test with Levene’s test for variance correction. Data are expressed as the mean ± SD of a total of three replicates.

### 2.3. Sample Preparation and RNA Extraction

Embryos destined for RNA extraction were washed three times in PBS and transferred to a microtube in a volume of 5 µL of the same medium. Samples were snap frozen by immersing the tubes in liquid nitrogen and then stored at −80 °C until the extraction process was resumed. RNA extraction was performed with the RNeasy Plus Micro Kit (Qiagen, Hilden, Germany) according to the manufacturer’s instructions. The quality and quantity of the isolated total RNA was measured using a Bioanalyzer 2100 (Agilent, Santa Clara, CA, USA) and a Nanodrop 2000 (ThermoFisher Scientific, Madrid, Spain). The RNA quality obtained was considered adequate for the performance of the experiment with an RNA integrity number always higher than 8.

### 2.4. Microarray Hybridization

The procedure applied to the samples was previously described [27] using the GeneChip 3′ IVT Pico Reagent kit according to the manufacturer’s instructions. Briefly, the same amount of total RNA isolated (650 pg) from each pool was reverse transcribed to synthesize single-stranded cDNA and finally converted to double-stranded DNA prior to the in vitro transcription process for the amplification of complementary RNA (cRNA) by the Eberwine method [28], thus ensuring enough material to continue the process. Once amplified, the cRNA was again retrotranscribed to obtain ds-cDNA, and after purification, it was fragmented and labeled with biotin. In the different steps required by the protocol, the quality and quantity of the cRNA or cDNA was duly measured using a Bioanalyzer 2100 and a Nanodrop 2000. Then, biotin-labeled cDNA fragments (4.5 µg) were hybridized to the GeneChip Porcine Genome Array from Affymetrix (Santa Clara, CA, USA) using the GeneChip Hybridization, Wash and Stain kit (P/N 90720; Affymetrix). This array chip provides extensive coverage of the porcine transcriptome and able to screen 23,256 transcripts representing 20,201 genes. Finally, the hybridized array chips were scanned using the Affymetrix GeneChip scanner GCS3000 and the collected data were processed by the Affymetrix Expression Command Console.

### 2.5. Microarray Data Analyses

The microarray analyses based on statistics and the interpretation of the biological results were performed with the software Partek Genomics Suite and Partek Pathways (Partek Incorporated, St. Louis, MO, USA). Once the data for each microarray were obtained, the first step was to normalize the intensity values using the robust multichip average method [29]. This technique allowed us to obtain a normalized and corrected intensity value for each probe set of the different samples. A principal component analysis (PCA) was initially performed, to analyze the degree of variation in the set of transcripts for each experimental group. Next, to obtain the list of differentially expressed genes (DEG) in each pairwise comparison, one-way ANOVA was performed, and the *p* value was adjusted using the Benjamini–Hochberg procedure (FDR). Genes with an adjusted *p* value of less than 0.05 and a minimum fold change of |1.5| were considered differentially expressed. Finally, the DEG lists were subjected to gene ontology term and pathway enrichment analysis based on the Kyoto Encyclopedia of Genes and Genomes (KEGG) database [30], both using the Fisher’s exact test.

### 2.6. Validation of Results by q-PCR

For validation of the microarray results, a total of 12 DEG were analyzed by q-PCR. These genes were chosen based on their biological importance and their presence in the most altered pathways. The isolated RNA was retrotranscribed to cDNA using the Maxima H Minus First Strand cDNA Synthesis Kit (Thermo Fisher Scientific, Waltham, MA, USA) according to the manufacturer’s instructions with the following thermocycling profile: 25 °C for 10 min, 50 °C for 15 min and 85 °C for 5 min.

The different primers were designed using Primer3plus software [31] and the NCBI [32] and Ensembl [33] databases. Before the experiment, each primer pair efficiency was obtained according to the equation E = 10[−1/slope], where the slope was calculated from the regression line obtained from 5 serial dilutions. The sequences of the primers used in the experiment and their calculated efficiencies are shown in Table 1.

qPCR was performed with iTaq Universal SYBR Green Supermix (Applied Biosystems, Foster City, CA, USA) with a volume of 10 µL per reaction including a cDNA amount of 2 µL and a primer concentration of 500 nM. The reactions were performed in duplicate in a QuantStudio 5 Real-Time PCR System (Applied Biosystems, Foster City, CA, USA) with a thermocycling profile of 40 cycles, including a previous activation step of 50 °C for 2 min and 95 °C for 2 min. Cycles consisted of 5 s at 95 °C for denaturation and 30 s at 60 °C for annealing and extension. The final stage included an analysis of the melting curve verifying the presence of a single peak in the different PCRs. To confirm the specificity of the amplified products, some samples were subjected to electrophoresis on agarose gels to check the size of their amplicons. The geometric mean of the housekeeping gene expression RPL19 and GAPDH was used for data normalization. With the data obtained, the fold-changes for the different genes and experimental groups were calculated using the Pfaffl method [34]. After verification of the parametric distribution by the Shapiro–Wilk test, the fold-changes were analyzed by Student’s *t*-test together with Levene’s test for the correction of the homogeneity of variances using IBM SPSS 24.0 Statistics package (IBM, Chicago, IL, USA).

### 2.7. Experimental Design

**Figure 1:**
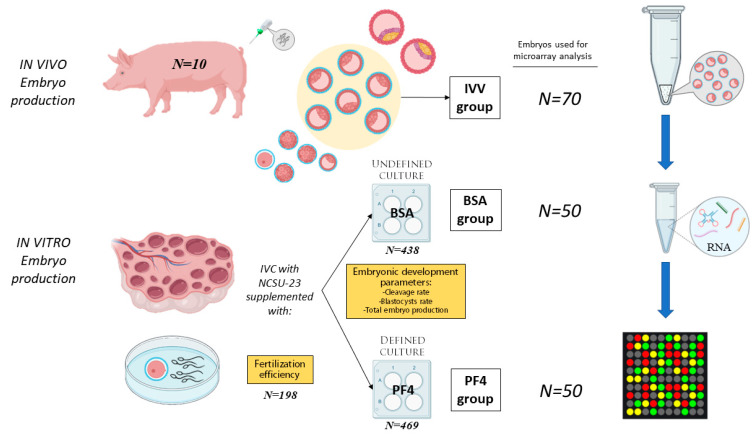
Representation of the experimental design conducted during the study. The samples consisted of pools of 10 blastocysts classified as ZP-covered and expanded. A total of three experimental groups were used, one of which consisted of surgically in vivo-obtained embryos (IVV group) and the other two consisted of embryos produced in vitro; the first one was cultured with undefined culture medium consisting of North Carolina State University (NCSU)-23 supplemented with 0.4 mg/mL bovine serum albumin (BSA group), and the second was cultured with a defined culture medium consisting of NCSU-23 supplemented with 0.3 mg/mL polyvinyl alcohol (PVA) and 100 ng/mL platelet factor 4 (PF4 group). The RNA was extracted from the pools and processed for microarrays. A total of 7 arrays were performed for the IVV group while 5 were performed for each in vitro group. IVC: in vitro culture

In this experiment, a total of 907 oocytes were matured and fertilized in vitro. The presumptive zygotes were cultured in NCSU-23 containing 0.3 mg/mL PVA and 100 ng/mL PF4 (defined medium; PF4 group; *N* = 469) or in NCSU-23 containing 0.4 mg/mL BSA (undefined medium; BSA group; *N* = 438) in a total of three replicates. Blastocysts obtained from each culture system were evaluated, and those classified as expanded were chosen for the microarray experiment (50 blastocysts for each in vitro group). In addition, a total of 70 in vivo-derived blastocysts classified at the same stage of development (IVV group) were also subjected to microarray analysis. The selected blastocysts were grouped into pools of 10 blastocysts, performing one array for each embryo pool, thus resulting in five microarrays for the PF4 group, another five for the BSA group and seven for the IVV group. Figure 1 schematically represents the experimental design conducted.

## 3. Results

### 3.1. Results of In Vivo and In Vitro Embryo Production

All sows used to obtain the in vivo-derived embryos had presence of embryos at day 6 after insemination. The number of corpora lutea of the sows ranged from 12 to 25, with a recovery rate of 91.6%, giving a total of 212 structures recovered. Of these, 8 (3.8%) were unfertilized oocytes or degenerated embryos, 41 (19.3%) were morulae and 163 (76.9%) were considered blastocysts. A total of 70 blastocysts classified as expanded were selected for this experiment while the rest were used for other experiments.

Regarding in vitro embryo production, the mean fertilization efficiency obtained from the three replicates (*N* = 198) was 41.9% ± 2.3%. The cleavage rates were similar for the PF4 and BSA groups (56.7% ± 5.6% and 61.6% ± 8.5%, respectively). The blastocyst rate on day 5 was higher (*p* < 0.004) for PF4 (48.1% ± 11.0%) than for BSA (32.1% ± 12.9%), while on day 6, there were no differences between groups (62.6% ± 9.3% and 52.3% ± 14.9% for the PF4 and BSA groups, respectively). Additionally, there were no differences in the total embryo production (35.6% ± 6.6% and 33.0% ± 12.3%) for the PF4 and BSA groups, respectively.

### 3.2. Transcriptional Profile

**Figure 2:**
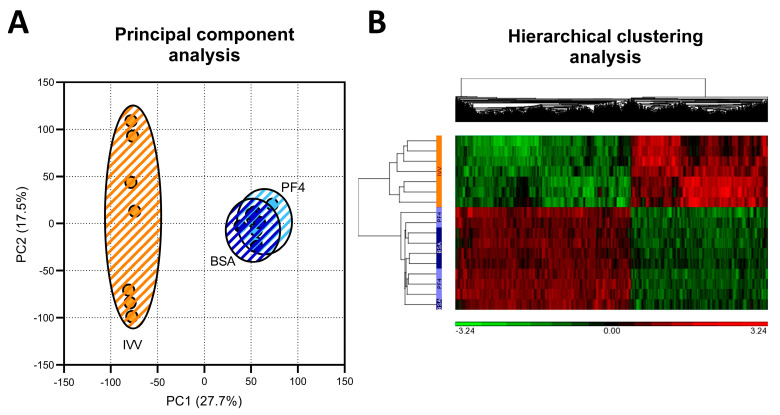
Distribution of the analyzed samples according to unsupervised analysis. (**A**) Principal component analysis obtained from the microarray results. (**B**) Hierarchical clustering analysis comparing the differentially expressed genes (|FC|</>1.5 and adjusted *p* value < 0.05) for the three experimental groups.

By the use of microarray technology, a total of 24,123 transcripts were detected, of which 16,462 were annotated as known genes and used for subsequent analyses. The PCA grouped the samples from the three experimental groups into two different populations, clearly separating the samples according to whether the embryos were originated in vivo or in vitro and therefore keeping the samples from the two in vitro groups together (Figure 2A). This pattern was confirmed by hierarchical clustering analysis (Figure 2B).

**Figure 3:**
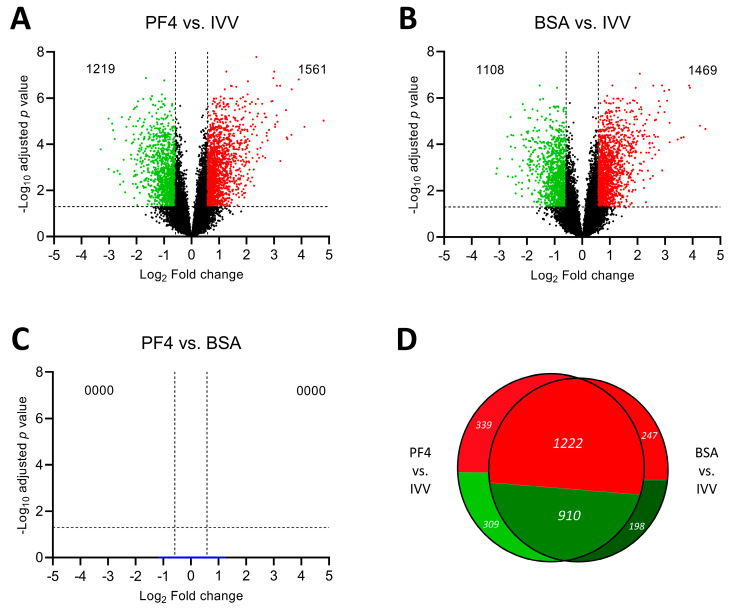
Differentially expressed genes (DEGs) represented by a volcano plot of each pairwise comparison. (**A**) Comparison between in vitro embryos cultured in defined medium (PF4: platelet factor 4) versus in vivo-derived embryos (IVV). (**B**) Comparison between in vitro embryos cultured in undefined medium (BSA: bovine serum albumin) versus IVV embryos. (**C**) Comparison between PF4 and BSA embryos. (**D**) Venn diagram comparison showing the cross-analysis of the DEG obtained from each in vitro group comparison against the IVV group.

The expression of the total set of transcripts analyzed is represented in volcano plots for each pairwise comparison made between the three experimental groups (Figure 3). When the PF4 group was compared to the IVV group, a total of 2780 DEG were determined (PF4 list), of which 1219 were downregulated while 1561 were upregulated (Figure 3A). On the other hand, when the groups compared were the BSA group versus the IVV group, a total of 2577 DEG were obtained (BSA list), among which 1108 were downregulated and 1469 were upregulated (Figure 3B). Then, the cross-analysis of the two lists revealed they shared 2132 DEG. These differences are graphically represented by a Venn diagram in Figure 3D. Finally, the comparison between the two in vitro groups (PF4 versus BSA), did not show any DEG (Figure 3C).

### 3.3. Gene Ontology and Pathway Enrichment Analysis

**Figure 4:**
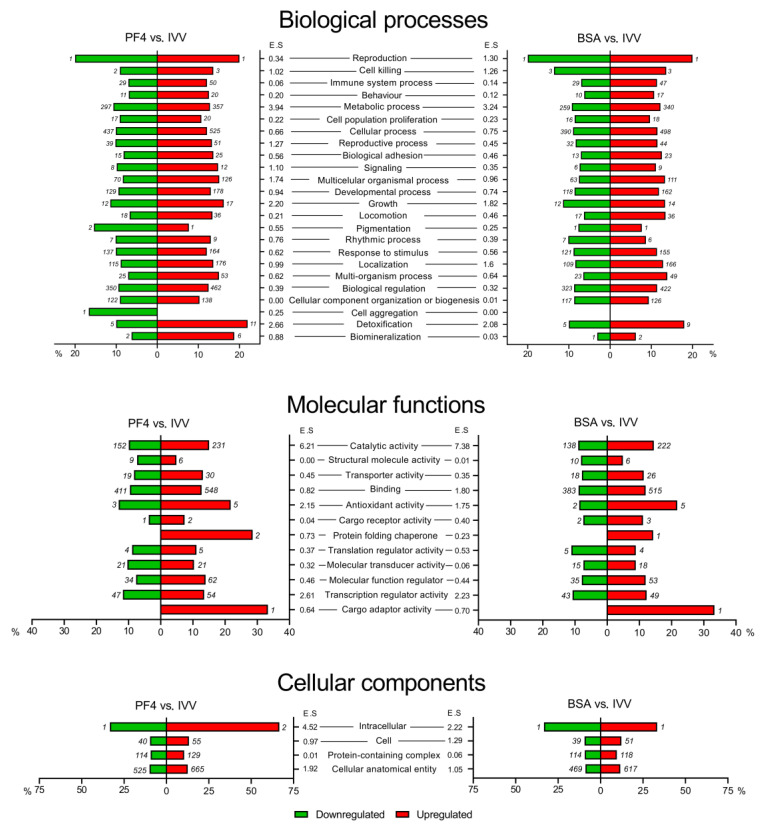
Classification of differentially expressed genes (DEG) organized by gene ontology (GO)-terms. The lists of DEG were obtained after comparing the in vitro embryos cultured in defined media (PF4: platelet factor 4) versus the in vivo-derived embryos (IVV), and the in vitro embryos cultured in undefined media (BSA: bovine serum albumin) versus the IVV embryos. In each forest plot, the *X*-axis represents the percentage of DEG out of the total number of genes belonging to each category. Green bars represent the percentages of downregulated DEG, while the red bars indicate the percentages of upregulated DEG. Next to each bar, the number of upregulated (red bar) or downregulated (green bar) DEG, belonging to that GO-term is indicated. ES: enrichment score.

The different GO terms obtained with the two lists of DEG (PF4 and BSA lists) were classified into the main categories of biological processes, molecular functions, and cellular components. These three major categories were broken down into second level GO terms, all of which are shown in Figure 4. For the category of biological processes, the functions with the highest percentage of affected genes in both lists (PF4 and BSA) were reproduction (40.0% and 40.0%), detoxification (32.0% and 28.0%), growth (27.6% and 24.8%) and cell killing (22.7% and 27.3%), respectively. On the other hand, for the category of molecular function, the antioxidant (34.8% and 30.4%) and cargo adaptor activity (33.3% and 33.3%) terms were those with a higher percentage of altered genes while in the category of cellular component, the intracellular term (100% and 66.7%) had the highest percentage of DEG for the PF4- and BSA-lists, respectively.

**Figure 5:**
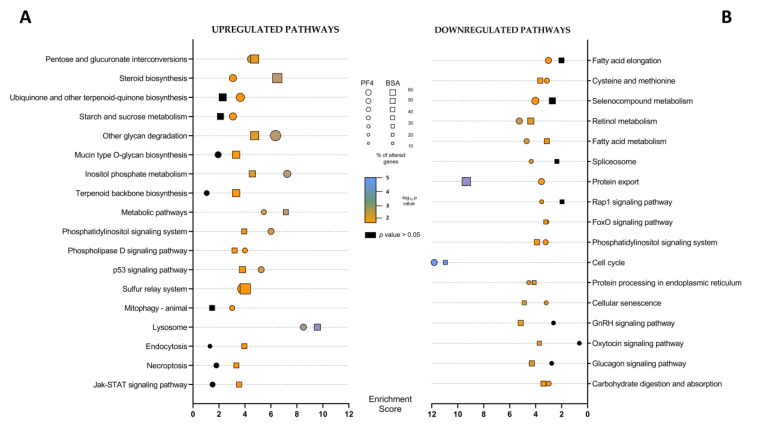
Representation of the different pathways considered altered (*p* value < 0.05) obtained from the (**A**) upregulated differentially expressed genes (upregulated pathways) and from (**B**) the downregulated differentially expressed genes (downregulated pathways). The enrichment values for each pathway are indicated by a circle in the case of the comparison between in vitro embryos cultured in defined medium (PF4: platelet factor 4) and in vivo-derived embryos (IVV) and by a square in the case of the comparison between in vitro embryos cultured in undefined medium (BSA: bovine serum albumin) and IVV embryos. The size of the symbol indicates the percentage of affected genes out of the total number of genes belonging to that pathway. The color of the symbol indicates the p-value of each pathway.

For the determination of the KEGG pathways, each DEG list was divided according to their positive or negative fold-change value, thus allowing the detection of pathways significantly altered for up- or downregulated transcripts. Only those pathways with a *p* value < 0.05 were considered significantly affected. In addition, those pathways that met these criteria but were unrelated to the biological samples analyzed in this experiment, were discarded. There were 13 and 15 upregulated KEGG pathways on the PF4 and BSA lists, respectively. Of these pathways, 10 were common on both lists (Figure 5A and Supplementary Table S1). Regarding the downregulated KEGG pathways, 14 were identified in the PF4-list and 13 in the BSA-list, sharing 10 of them (Figure 5B and Supplementary Table S2). Supplementary information regarding the different genes affected belonging to each pathway is given in Supplementary Tables S3 and S4.

**Figure 6:**
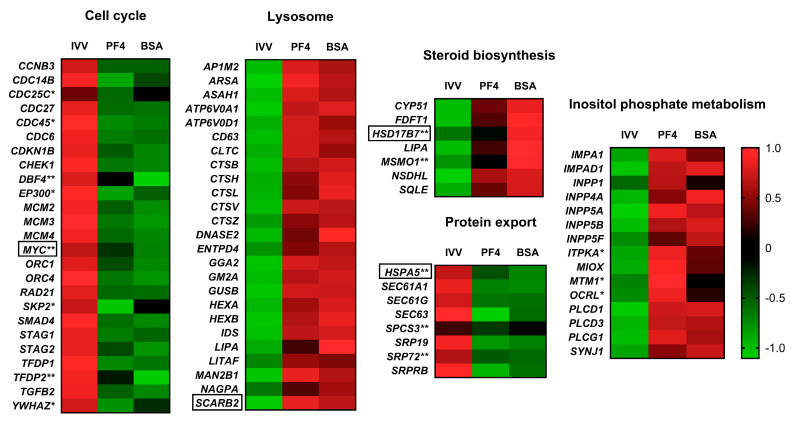
Heat maps of the most prominent pathways representing the expression of each differentially expressed gene in each experimental group: in vivo-derived embryos (IVV), in vitro embryos cultured in defined medium (PF4: platelet factor 4) and in vitro embryos cultured in undefined medium (BSA: bovine serum albumin). * Indicates a gene considered differentially expressed in the case of the PF4 versus IVV comparison but not in the BSA versus IVV comparison. ** Indicates a gene considered differentially expressed in the BSA versus IVV comparison but not in the PF4 versus IVV comparison. Genes without any symbol indicate that they were considered differentially expressed in both comparisons. Genes surrounded by a box were selected for validation by q-PCR.

Of the different affected pathways, those that presented a higher enrichment score in both lists were the lysosome pathway (enrichment scores of 8.5 and 9.6, respectively) for upregulated DEG and the cell cycle pathway (enrichment score of 11.8 and 11.0 respectively), for downregulated DEG. In addition, some altered pathways showed higher enrichment scores and percentages of DEG in the BSA-list than in the PF4-list, such as the protein export and steroid biosynthesis pathways (Figure 5 and Supplementary Tables S1 and S2). In contrast, the inositol phosphate metabolism was more affected in PF4 than in the BSA list (Figure 5A and Table S1). The intensity of DEG expression belonging to the most representative altered pathways is shown in different heat maps in Figure 6.

**Figure 7:**
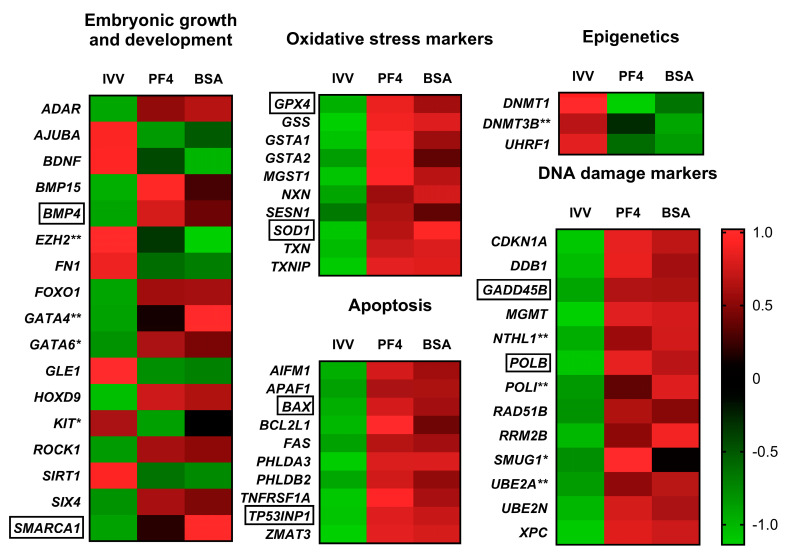
Heat maps grouping differentially expressed genes by specific functions at the embryonic level or as markers of damage for the three experimental groups: in vivo-derived embryos (IVV), in vitro embryos cultured in defined medium (PF4) and in vitro embryos cultured in undefined medium (BSA). * Indicates a gene considered differentially expressed in the case of the PF4 (PF4: platelet factor 4) versus IVV comparison but not in the BSA (BSA: bovine serum albumin). versus IVV comparison. ** Indicates a gene considered as differentially expressed in the BSA versus IVV comparison but not in the PF4 versus IVV comparison. Genes without any symbol indicate that they were considered differentially expressed in both comparisons. Genes surrounded by a box were selected for validation by q-PCR.

Some DEG related to different processes such as oxidative stress damage, apoptosis, DNA damage, alteration in processes related to epigenetics and other genes related to cell growth and differentiation, were also highlighted as embryo quality and developmental competence markers (Figure 7).

### 3.4. Validation by qPCR

**Figure 8:**
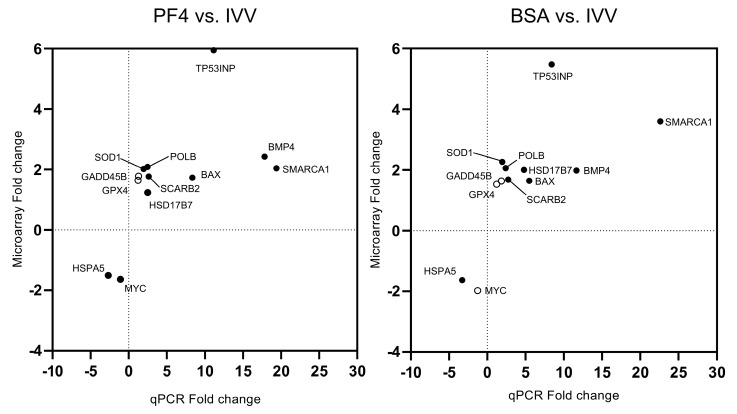
Comparison of the fold changes obtained by microarray and q-PCR analyses for the different validated genes in the experiment. The black circles represent genes where the significance obtained with the q-PCR was accordingly to those obtained by the microarray. White circles represent genes where the significance obtained with q-PCR differs from those obtained by the microarray.

All genes subjected to the validation process showed similar fold change patterns in q-PCR with respect to the microarray results (Figure 8). Of the 12 genes, the significance was identified accordingly to the microarray results for 10 genes of the PF4- versus the IVV-list and for 9 in the case of the BSA- versus the IVV-list.

## 4. Discussion

This work analyzed for the first time the transcriptome of in vitro-produced pig embryos cultured in a fully defined medium using microarray technology, which provides a comprehensive understanding of the blastocyst transcriptome.

Of the more than 24,000 transcripts analyzed, none of them showed significant differences between embryos cultured in PF4 against those cultured in BSA. These results demonstrate the ability of PF4 as a substitute for BSA, as has been previously demonstrated [20] and confirmed in this study in embryonic production terms. Although the transcriptome patterns were similar in both IVP groups, it is interesting to mention some pathways that appeared to be more altered in one of the experimental groups compared to the in vivo-derived embryos. For instance, the steroid biosynthesis pathway presented a higher enrichment score and percentage of overexpressed genes in the case of BSA (6.5 and 46.7%) compared to PF4 (3.1 and 33.3%). DEG found exclusively in the BSA group included *HSD17B7* and *MSMO1*, which are involved in cholesterol synthesis [35,36]. The upregulation of this pathway could be related to the ability of BSA to bind fatty acids and steroids [37,38], releasing them into the culture medium. This phenomenon has been described during the in vitro maturation of bovine oocytes [39], where supplementation with BSA increased the amount of total lipids in oocytes with respect to those in vivo-derived. This fact could easily explain how embryos cultured in BSA would increase the expression of genes encoding enzymes that metabolize these types of compounds.

Another pathway with a higher enrichment value and percentage of downregulated genes in the BSA group (9.3 and 40%) than in the PF4 group (3.5 and 25%), was the protein export pathway. Among the genes affected, the most notable was *HSPA5*, also known as *GRP78*, which was significantly downregulated in the BSA group but not in the PF4 group. This gene has a primordial role in the protein folding process, and is able to identify misfolded proteins to ensure their correction or lead to their degradation [40,41]. Thus, the repression of this gene suggests an alteration in the embryos developed in BSA that would disturb this basic process for the normal development of the cells. Further studies are required to determine why this pathway is less altered in IVP embryos cultured in PF4.

In contrast, embryos cultured in PF4 showed a higher activation of inositol phosphate metabolism pathway in terms of the enrichment score and percentage of affected genes (7.3 and 33.3%) compared to those embryos cultured in BSA (4.6 and 26.7%). The differences observed between both in vitro culture groups could be explained by the presence of small traces of basic metabolites of these pathways, such as myo-inositol, in BSA. The beneficial effects of myo-inositol as a supplement in a defined medium for bovine embryos have been described. However, when myo-inositol was combined with BSA these effects were no longer appreciated [42], suggesting the presence of this compound in BSA. With this as a premise, it becomes interesting to study the effects of inositol metabolites as supplements for culture medium with PF4 to determine if the alterations in this pathway would be reduced, bringing their status closer to that of the in vivo-derived embryos. All these minimal transcriptomic differences between both culture media did not influence the blastocysts formation rates at the end of IVC period. However, in agreement with our previous study [20], the early blastocyst formation rate (day five) was slightly increased in the PF4 medium. Further research is needed to verify if these differences at day five are due to major alterations in the transcriptome between both groups.

The comparison of the in vitro groups with the in vivo-derived embryos revealed that the differences in the expression patterns were due to the embryo origin with samples cultured in BSA and PF4 clearly grouped together (as shown in the PCA and hierarchical clustering analyses). These results reflect how the in vitro embryo production system dramatically affects the transcriptome of the embryos. The most enriched pathway was the cell cycle pathway, which included a large number of downregulated genes. The altered genes belonging to this pathway include subunits 1 and 4 of the origin recognition complex (*ORC*), the components 2 and 4 of the mini-chromosome maintenance complex (*MCM*) and different cycle division genes (*CDCs*) such as *CDC6*. ORCs encode proteins that bind to DNA during the G1 phase to signal the starting site for replication. Then, the ORCs recruit other proteins, such as CDC6, that in turn recruit the MCM, thus forming the prereplication complex [43]. Another downregulated gene involved in the cell cycle (only BSA embryos) was the *MYC* gene, which is related to the maintenance of the pluripotent state [44]. Another remarkable repressed gene, *SMAD4*, has been demonstrated to be essential for embryonic development in bovines [45] and mice [46]. This gene is also an important effector of the transforming growth factor pathway [47], and one of its components, *TGFB2*, also downregulated which is related to cell differentiation [48]. The fact that many of the components involved in the cell cycle pathway were downregulated in the IVP embryos suggests a reduction of this process, which may imply important developmental consequences.

Although not included in the cell cycle pathway, certain genes related to embryonic development and growth were altered in the IVP embryos. Some of these genes such as *BMP4*, *ADAR*, *KIT* (only in PF4) and *EZH2* (only in BSA), have been previously reported as altered in porcine IVP embryos [4]. In the present study, other important genes including the zing finger transcription factors GATA4 (only in BSA) and *GATA6* (only in PF4) related to preimplantation development [49], *SMARCA1*, which is involved in the control of cell growth [50], *ROCK1*, which is required for early embryonic development in both murine [51] and porcine [52] species, and *FN1*, which plays important roles during gastrulation [53], were altered in the IVP embryos. It is noteworthy to mention the repression of BDNF, a growth factor whose exogenous supplementation enhances in vitro oocyte development and subsequent blastocyst formation in pigs [54] and buffalos [55]. The altered expression of this variety of genes with respect to the in vivo-derived embryos reveals the abnormalities derived from the IVC and how they would justify the developmental delays and alterations in quality observed in the cultured embryos [56].

Another important group of DEG includes those involved in epigenetics, which are able to regulate gene expression, thus conditioning embryonic development [57]. In this study, we found that the expression of the *DNMT1* gene and its cofactor UHRF1 was reduced in both in vitro groups. However, the gene *DNMT3B*, which is involved in the novo DNA methylation in human blastocysts prior to implantation [58] was only downregulated in the in vitro embryos cultured with BSA. This finding suggests some small differences in the epigenetic processes between both in vitro groups; however, further research is needed to reach precise conclusions.

Regarding the pathways formed by overexpressed genes, the most outstanding is the lysosome pathway. The genes that constitute this pathway and that were altered form different components necessary for the functioning of lysosomes such as different cathepsins (CTSB, CTSH, CTSL, CTSV and CTSZ), glycosidases (HEXA, HEXAB, GUSB and MAN2B1), lipases (LIPA) and nucleases (DNASE2), among others, involved in the degradation of different molecules inside lysosomes [59]. Lysosome activity is closely related to the autophagy process [60], which is vital for development [61]. However, the activation of this pathway observed in IVP embryos might be a response to the stress caused by different harmful conditions, such as starvation or oxidative stress [61]. The IVP embryos also showed overexpression of several genes belonging to the metabolic pathway, as has been previously described by Bauer et al., [3]. Many authors have indicated that a relatively low metabolism would be associated to a higher embryonic viability [62,63] or at least a more balanced metabolism, which would not tend to be at extreme ranges [64].

Another group of genes that were significantly altered in both PF4 and BSA embryos compared to in vivo-derived were related to oxidative stress. As has already been highlighted in several previously published articles, IVC conditions dramatically aggravate the stress suffered by embryos [65,66]. This situation is reflected in this study where different genes related to oxidative stress were upregulated in IVP embryos, including essential genes involved in the protection of cells against free radicals such as *GSS*, *GPX4*, *GSTA*, *MGST1* [67], *TXN*, *TXNIP*, *NXN* [68], *SOD1* [69] and *SESN1* [70]. One of the main consequences of this overexposure to different types of free radicals is the damage that they cause to DNA [71], which has been confirmed in both oocytes [72] and embryos [73,74,75]. In that sense, in this study, we observed how, independent of the culture type, IVP embryos overexpressed genes involved in DNA repair processes. Of this group of genes, the one that showed the greatest differences with respect to the IVV was *MGMT*, depicting fold change values above 8 in both the BSA and PF4 groups. This gene encodes for a protein responsible for accepting alkyl groups from damaged nucleotides and reestablishing their functionality [76], which has been demonstrated as a typical injury triggered by oxidative damage [77]. Other interesting genes involved in DNA repair were *GADD45B* [78], *DDB1* [79] and *POLB*, which repair short patch base excisions [80]. Although DNA repair is a physiological mechanism necessary for the process of embryonic development and to avoid cell arrest [81], the differences observed in IVP embryos indicate damage at this level, probably induced by this increased oxidative stress. If the cell is unable to repair DNA lesions, then apoptotic pathways could be activated to eliminate damaged cells [82]. In the present study, overexpression of certain genes related to this process was observed. Of these proapoptotic altered genes, the *TP53INP1* stands out and presents fold change values higher than five in PF4 and BSA embryos. This proapoptotic gene, induces apoptosis and cell cycle arrest in cells subjected to different types of stress, including oxidative stress agents [83,84]. Other genes related to the apoptotic process were also overexpressed such as *FAS*, which has been widely used as an indicator of apoptosis in blastocysts of various species [85,86,87], *AIFM1*, a mitochondrial effector of apoptotic cell death [88] and *BAX*, whose expression levels would influence oocyte and embryo survival [89].

Taken together, this cascade of events impairs in vitro embryo development, reducing the IVP yield, with a high percentage of embryos that do not reach the adequate stage of development [90], and a reduced quality of the embryos that also contributes to the low rates of embryo implantation performed with produced in vitro embryos [5,6,7].

## 5. Conclusions

The chemically defined medium, NCSU-23 supplemented with PVA and PF4 can sustain porcine embryo production with the same efficiency as NCSU-23 BSA, thereby providing blastocysts of similar quality in terms of the gene expression profile. This study also demonstrates the differences in the transcriptome between in vitro- and in vivo-derived blastocysts, confirming that there is still much work to be done to improve IVP; however, this arduous task is expected to be facilitated by the use of defined culture media.

## Figures and Tables

**Table 1:** Sequences of the primers used for validation of the microarray results by q-PCR.

Gene	ID	Forward (5′→3′)	Reverse (5′→3′)	Size	Efficiency	R^2^
*BAX*	NM_214285.1	GCTGACGGCAACTTCAACTG	GCGTCCCAAAGTAGGAGAGG	202	90.3	0.993
*BMP4*	NM_001101031	TACATGCGGGATCTTTACCG	AAGCAGAGTTTTCGCTGGTC	172	110.1	0.995
*GADD45B*	XM_005654701	ACCCTCATCCAGTCGTTTTG	GCTTTTCCAGGCATCTGTGT	171	104.5	0.998
*GPX4*	NM_214407.1	GAGCTTTAGCCGCCTGTTC	GGTACTTGTCCAGGTTCACCA	176	105.7	0.997
*HSD17B7*	NM_001185137	AGCGATTCATGTGTTCTCCA	GGATGTCCTCAAGGCTGAAA	218	99.1	0.994
*HSPA5*	XM_001927795	GGAAACTGCTGAGGCTTATTTG	TCCCCTTCCCTCTTATCCAG	189	102.7	0.993
*MYC*	NM_001005154	TCGGACTCTCTGCTCTCCTC	GCTGCCTCTTTTCCACAGAA	157	102.0	0.997
*POLB*	XM_005657652	GTTTGCCAGCTTCCCAGTAA	CCACAGGACGGATTGTGTATT	193	98.6	0.992
*SCARB2*	NM_001244155	TGGTTTTCCCAGTGATGTATCT	CAGGTGAAGATCAGACCGAAG	154	98.5	0.996
*SMARCA1*	XM_003135362	CCTCCAAAACAGCCAAATGT	GGTGTAAGAGGTTCAGCTCCA	191	94.6	0.995
*SOD1*	NM_001190422.1	GGATCAAGAGAGGCACGTTG	CTGCCCAAGTCATCTGGTTT	159	91.8	0.999
*TP53INP1*	XM_001925224	CCAGGTAGTCCCAGAGTGGA	TAAGATTTTGGCGACGAAGG	184	91.4	0.999
*GAPDH*	NM_001206359	ATCACTGCCACCCAGAAGAC	AGATCCACAACCGACACGTT	194	96.6	0.999
*RPL19*	XM_003131509	AGCCTGTGACTGTCCATTCC	AGTACCCTTCCGCTTACCGA	95	99.3	0.998

## Supplementary Materials

The following are available online at https://www.mdpi.com/article/10.3390/ani11051414/s1, Table S1: Results of the enrichment score analyses for the different overexpressed pathways, Table S2: Results of the enrichment score analyses for the different underexpressed pathways, Table S3: Lists of the upregulated differentially expressed genes belonging to the different altered pathways, Table S4: Lists of the downregulated differentially expressed genes belonging to the different altered pathways.
