# Supplementary material for: Transcriptional Profiling of Porcine Blastocysts Produced In Vitro in a Chemically Defined Culture Medium
Source: Animals (Basel). 2021 May 14;11(5):1414. doi: 10.3390/ani11051414 (PMC8156047; doi:10.3390/ani11051414)
Supplement: Supplementary file 1 [file animals-11-01414-s001.zip › animals-1195921-supplementary.pdf]

Supplementary files

Transcriptional profiling of porcine blastocysts produced *in vitro* in a chemically defined culture medium

Josep M. Cambra [1,2], Emilio A. Martinez[1,2], Heriberto Rodriguez-Martinez[3], Maria A. Gil[1,2*], Cristina Cuello[1,2]

[1]Department of Medicine and Animal Surgery, Faculty of Veterinary Medicine, International Excellence Campus for Higher Education and Research "Campus Mare Nostrum", University of Murcia, 30100, Murcia, Spain; josepmiquel.cambra@um.es; emilio@um.es; mariagil@um.es; ccuello@um.es

[2]Institute for Biomedical Research of Murcia (IMIB-Arrixaca), Campus de Ciencias de la Salud, Carretera Buenavista s/n, 30120 El Palmar, Murcia, Spain.

[3]Department of Biomedical & Clinical Sciences (BKV), BKH/Obstetrics & Gynaecology, Faculty of Medicine and Health Sciences, Linköping University, SE-58185 Linköping, Sweden; heriberto.rodriguez-martinez@liu.se

* Correspondence: mariagil@um.es

Supplementary Table S1: Results of the enrichment score analyses for the different over-expressed pathways

Pathway Name (UPREGULATED)	Pathway ID	Enrichment score difference	PF4 vs IVV			BSA vs IVV		
			Enrichment Score	Enrichment p-value	% genes in pathway	Enrichment Score	Enrichment p-value	% genes in pathway
Pentose and glucuronate interconversions	4	-0.3	4.5	0.0114	45.5	4.8	0.0086	45.5
Steroid biosynthesis	12	-3.4	3.1	0.0462	33.3	6.5	0.0015	46.7
Ubiquinone and other terpenoid-quinone biosynthesis	14	1.4	3.6	0.0261	44.4	2.3	0.1010 (n.s)	33.3
Starch and sucrose metabolism	40	1.0	3.1	0.0462	33.3	2.1	0.1205 (n.s)	26.7
Other glycan degradation	42	1.6	6.4	0.0017	54.5	4.8	0.0086	45.5
Mucin type O-glycan biosynthesis	43	-1.4	1.9	0.1443 (n.s)	26.7	3.3	0.0360	33.3
Inositol phosphate metabolism	53	2.7	7.3	0.0007	33.3	4.6	0.0103	26.7
Terpenoid backbone biosynthesis	79	-2.3	1.1	0.3474 (n.s)	20.0	3.3	0.0360	33.3
Metabolic pathways	87	-1.7	5.5	0.0043	16.9	7.1	0.0008	16.5
Phosphatidylinositol signaling system	127	2.1	6.0	0.0025	28.3	3.9	0.0193	23.3
Phospholipase D signaling pathway	129	0.8	4.0	0.0183	23.1	3.2	0.0405	20.5
p53 signaling pathway	133	1.4	5.3	0.0052	29.5	3.8	0.0222	25.0
Sulfur relay system	135	-0.2	3.8	0.0215	60.0	4.0	0.0179	60.0
Mitophagy - animal	138	1.5	3.0	0.0490	23.1	1.5	0.2288 (n.s)	17.3
Lysosome	141	-1.1	8.5	0.0002	28.7	9.6	6.90E-05	28.7
Endocytosis	142	-2.6	1.3	0.2696 (n.s)	15.8	4.0	0.0191	19.0
Necroptosis	153	-1.5	1.8	0.1648 (n.s)	18.0	3.3	0.0352	20.2
Jak-STAT signaling pathway	183	-2.0	1.5	0.2202 (n.s)	17.2	3.6	0.0286	20.7

The enrichment score difference shows the subtraction of the enrichment score of each pathway obtained from the list of upregulated DEGs resulted from the comparison of the PF4 group versus the IVV group and the list of upregulated DEGs obtained by comparing the BSA group versus the IVV group. The % of genes in pathway describes the percentage of genes altered in each of the lists of upregulated DEGs out of the total number of genes belonging to that pathway, according to the KEGG database. n.s: non-significant.

Supplementary Table S2: Results of the enrichment score analyses for the different under-expressed pathways.

Pathway Name (DOWNREGULATED)	Pathway ID	Enrichment score difference	PF4 vs IVV			BSA vs IVV		
			Enrichment Score	Enrichment p-value	% genes in pathway	Enrichment Score	Enrichment p-value	% genes in pathway
Fatty acid elongation	9	1.0	3.0	0.0493	25.0	2.0	0.1336 (n.s)	18.8
Cysteine and methionine metabolism	23	-0.5	3.1	0.0439	18.9	3.6	0.0262	18.9
Selenocompound metabolism	36	1.3	4.0	0.0179	33.3	2.7	0.0664 (n.s)	25.0
Retinol metabolism	77	0.9	5.3	0.0052	28.0	4.4	0.0126	24.0
Fatty acid metabolism	90	1.6	4.7	0.0092	22.0	3.1	0.0434	17.1
Spliceosome	105	2.0	4.3	0.0131	16.3	2.4	0.0941 (n.s)	12.2
Protein export	107	-5.8	3.5	0.0287	25.0	9.3	8.82E-05	40.0
Rap1 signaling pathway	118	1.6	3.5	0.0290	14.7	2.0	0.1402 (n.s)	11.2
FoxO signaling pathway	126	-0.1	3.1	0.0432	14.7	3.2	0.0402	13.7
Phosphatidylinositol signaling system	127	-0.7	3.2	0.0396	16.7	3.9	0.0204	16.7
Cell cycle	131	0.9	11.8	7.45E-06	24.7	11.0	1.75E-05	22.5
Protein processing in endoplasmic reticulum	140	0.4	4.5	0.0109	15.7	4.1	0.0162	14.0
Cellular senescence	154	-1.7	3.2	0.0413	14.3	4.9	0.0077	15.2
GnRH signaling pathway	216	-2.5	2.6	0.0724 (n.s)	15.5	5.1	0.0058	19.0
Oxytocin signaling pathway	225	-3.1	0.6	0.5275 (n.s)	9.2	3.7	0.0244	14.3
Glucagon signaling pathway	226	-1.5	2.8	0.0632 (n.s)	15.4	4.3	0.0137	16.9
Carbohydrate digestion and absorption	247	-0.4	3.0	0.0499	21.7	3.4	0.0334	21.7

The enrichment score difference shows the subtraction of the enrichment score of each pathway obtained from the list of downregulated DEGs resulted from the comparison of the PF4 group versus the IVV group and the list of downregulated DEGs obtained by comparing the BSA group versus the IVV group. The % of genes in pathway describes the percentage of genes altered in each of the lists of downregulated DEGs out of the total number of genes belonging to that pathway, according to the KEGG database. n.s: non-significant.

Supplementary Table S3: Lists of the upregulated differentially expressed genes belonging to the different altered pathways.

Pathway Name (UPREGULATED)	Pathway ID	Upregulated DEGs only in PF4 vs IVV	Upregulated DEGs in both comparisons	Upregulated DEGs only in BSA vs IVV
Pentose and glucuronate interconversions	4		*CRYL1, DHDH, GUSB, SORD, UGP2*	
Steroid biosynthesis	12		*CYP51, FDFT1, LIPA, NSDHL, SQLE*	*HSD17B7, MSMO1*
Ubiquinone and other tarpenoid-quinone biosynthesis	14	*VKORC1*	*COQ5, COQ6, NQO1*	
Starch and sucrose metabolism	40	*PGM1*	*GPI, GYG1, PYGL, UGP2*	*PYGM*
Other glycan degradation	42	*AGA*	*FUCA2, HEXA, HEXB, MAN2B1, MAN2C1*	
Mucin type O-glycan biosynthesis	42		*B4GALT5, GALNT12, GALNT2, GALNT3*	*GALNT7*
Inositol phosphate metabolism	53	*ITPKA, MTM1, OCRL*	*IMPA1, IMPAD1, INPP1, INPP4A, INPP5A, INPP5B, INPP5F, MIOX, PLCD1, PLCD3, PLCG1, SYNJ1*	
Terpenoid backbone biosynthesis	79		*FNTA, HMGCR, HMGCS1*	*FDPS, PCYOX1*
Metabolic pathways	87	*ACAA1, AGPAT4, AK2, ARG1, ATP6V1E1, ATP6V1F, BCAT1, CMBL, CYP3A46, GGT7, GLS, GMDS, GYG1, ITPKA, LTA4H, MRI1, MTM1, NDUFS8, NME1, OCRL, PC, PGM1, SMPD2*	*ABAT, ACADVL, ACOX2, ACSS3, ADA, ADSSL1, AGPAT3, AGPAT5, AGXT2, ALDH9A1, ALG13, AMACR, ASAH1, ATP5E, ATP6V0A1, ATP6V0D1, BLVRB, BPGM, CDA, CHPT1, CHST10, CMAS, CMPK1, CNDP1, COQ5, COQ6, COX6B, CPS1, CRYL1, CYP19A1, CYP19A2, CYP19A3, CYP51, DGKA, DHRS4, DLST, EPHX2, FDFT1, FOLH1B, FUT4, FUT8, GALNT12, GALNT2, GALNT3, GALT, GATB, GLUL, GPAT3, GPI, GRHPR, GSS, GULO, GUSB, HAAO, HEXA, HEXB, HMGCL, HMGCR, HMGCS1, HSD17B8, IDS, IMPA1, IMPAD1, INPP1, INPP4A, INPP5A, INPP5B, INPP5F, MAN1C1, MAN2A2, MDH1, ME1, MGAT4B, MOCS2, ND4L, NDUFA13, NDUFA3, NDUFA7, NFS1, NNT, NSDHL, NTPCR, OGDHL, P4HA2, PCCB, PFKP, PGLS, PHGDH, PLA2G16, PLCD1, PLCD3, PLCG1, PLD3, PNPLA2, POLR1D, POLR2I, POLR3GL, POMGNT2, PYGL, RFK, RRM2B, SAT2, SORD, SPR, SQLE, SUOX, SYNJ1, TKT, TST, TYMS, UGCG, UGP2, UPRT, UROD*	*ACO1, ACY1, ALDH18A1, COASY, CPOX, DCTD, FDPS, GALNT7, GGT6, HSD17B7, LIPT1, MSMO1, NADK2, NDUFA11, NUDT12, PYGM, ST6GALNAC6, TMEM5, TYR, UROS*
Phosphatidylinositol signaling system	127	*ITPKA, MTM1, OCRL*	*CALM3, DGKA, IMPA1, IMPAD1, INPP1, INPP4A, INPP5A, INPP5B, INPP5F, PIK3R2, PLCD1, PLCD3, PLCG1, SYNJ1*	
Phospholipase D signaling pathway	129	*AGPAT4, CXCL8*	*AGPAT3, AGPAT5, CYTH2, DGKA, FCER1G, GNA12, GNAS, KITLG, LPAR3, MTOR, PIK3R2, PLCG1, RALB, RRAS, SYK, TSC2*	
p53 signaling pathway	133	*TP53I3*	*APAF1, BAX, CDK2, CDKN1A, FAS, GADD45B, RRM2B, SERPINB5, SESN1, TSC2, ZMAT3*	
Sulfur relay system	135		*MOCS2, NFS1, TST*	
Mitophagy - animal	138	*BCL2L1, CITED2, GABARAPL1*	*CALCOCO2, FIS1, OPTN, PINK1, RHOT1, RRAS, SRC, TBC1D17, ULK1*	
Lysosome	141		*AP1M2, ARSA, ASAH1, ATP6V0A1, ATP6V0D1, CD63, CLTC, CTSB, CTSH, CTSL, CTSV, CTSZ, DNASE2, ENTPD4, GGA2, GM2A, GUSB, HEXA, HEXB, IDS, LIPA, LITAF, MAN2B1, NAGPA, SCARB2*	
Endocytosis	142	*EEA1, SMAD3*	*ARFGAP3, BIN1, CAV2, CHMP3, CLTC, CYTH2, EPS15, FGFR2, GRK5, HSP70.2, HSP70.2, HSPA2, HSPA8, LDLRAP1, PARD6G, RAB10, RAB11A, RAB11FIP2, SH3GLB2, SLA-8, SMURF2, SNF8, SRC, VPS45*	*CDC42, EHD1, LDLR, SLA-1, STAMBP, TFRC, VPS36*
Necroptosis	153	*HMGB1, STAT5A*	*AIFM1, BAX, CAMK2D, CHMP3, FAS, GLUL, JAK1, JAK3, PYGL, STAT1, STAT3, STAT6, TNFRSF1A*	*JAK2, PYGM, STAT5B, TLR4, TYK2*
Jak-STAT signaling pathway	183	*BCL2L1, STAT5A*	*CDKN1A, IL10RB, IL13RA1, IL17D, IL27RA, JAK1, JAK3, MTOR, PIK3R2, PTPN6, STAT1, STAT5B, STAT6*	*IL11RA, IL-6, JAK2, STAT3, TYK2*

Supplementary Table S4: Lists of the downregulated differentially expressed genes belonging to the different altered pathways.

Pathway name (DOWNREGULATED)	Pathway ID	Downregulated DEGs only in PF4 vs IVV	Downregulated DEGs in both comparisons	Downregulated DEGs only in BSA vs IVV
Fatty acid elongation	9	*EGFL8*	*ACAA2, HACD3, MECR*	
Cysteine and methionine metabolism	23	*MTAP, MTR*	*AHCYL2, CDO1, DNMT1, ENOPH1, MAT2B*	*DNMT3B, TAT*
Selenocompound metabolism	36	*MTR*	*PAPSS2, SEPHS1, SEPHS2*	
Retinol metabolism	77	*CYP26A1, CYP2C49*	*CYP1A1, CYP2C32, CYP2C33, CYP2C42*	
Fatty acid metabolism	90	*ACSL5, EGFL8*	*ACAA2, ACADM, ACSL3, FADS1, FADS2, HACD3, MECR*	
Spliceosome	105	*HNRNPA3, NCBP2, SRSF2*	*LSM6, RBM22, RBM25, RBMX, SF3B4, SNRNP40, SRSF1, SRSF5, SRSF6, TRA2B*	*BCAS2, EFTUD2*
Protein export	107		*SEC61A1, SEC61G, SEC63, SRP19, SRPRB*	*HSPA5, SPCS3, SRP72*
Rap1 signaling pathway	118	*F2R, KIT, MAPK14, MRAS, PRKD3*	*AKT3, BRAF, CALML4, EFNA1, GNAO1, LPAR1, MAP2K2, MAP2K6, P2RY1, PLCB3, PRKCB, RALA*	*CALM1*
FoxO signaling pathway	126	*EP300, MAPK14, SKP2, SOD2*	*AKT3, BRAF, CCNB3, CDKN1B, MAP2K2, PLK4, SIRT1, SMAD4, SOS1, TGFB2*	*KLF2, PRKAA1, PRKAB2*
Phosphatidylinositol signaling system	127	*ITPK1, MTMR7*	*CALML4, CDS2, ITPR1, PIK3C2A, PIP4K2A, PLCB3, PLCD4, PRKCB*	*CALM1, PPIP5K1*
Cell cycle	131	*CDC25C, CDC45, EP300, SKP2, YWHAZ*	*CCNB3, CDC14B, CDC27, CDC6, CDKN1B, CHEK1, MCM2, MCM3, MCM4, ORC1, ORC4, RAD21, SMAD4, STAG1, STAG2, TFDP1, TGFB2*	*DBF4, MYC, TFDP2*
Protein processing in endoplasmic reticulum	140	*EDEM3, HYOU1, MBTPS2, RNF185, STT3A*	*CANX, CAPN2, DDIT3, DNAJC10, EIF2S1, LMAN1, NSFL1C, PPP1R15A, SEC61A1, SEC61G, SEC63, STT3B, SVIP, TRAM1*	*HSPA5, MARCH6, SEC24D*
Cellular senescence	154	*MAPK14, SLC25A4*	*AKT3, CACNA1D, CALML4, CAPN2, CCNB3, CHEK1, ITPR1, MAP2K2, MAP2K6, MRAS, PPP1CC, SIRT1, TGFB2, TRPM7*	*CALM1, HUS1, LIN52, MYC*
GnRH signaling pathway	216	*MAP3K14*	*CACNA1D, CALML4, ITPR1, MAP2K2, MAP2K6, PLCB3, PRKCB, SOS1*	*CALM1, MAP3K3, PLA2G4A*
Oxitocyn signaling pathway	225		*CACNA1D, CACNB2, CALML4, GNAO1, ITPR1, MAP2K2, PLCB3, PPP1CC, PRKCB*	*CALM1, CD38, PLA2G4A, PRKAA1, PRKAB2*
Glucagon signaling pathway	226	*CREB3L2, EP300*	*AKT3, CALML4, CREB3L3, ITPR1, PLCB3, PPP4R3B, SIRT1, SLC2A1*	*CALM1, PRKAA1, PRKAB2*
Carbohydra digestion and absorption	247		*AKT3, CACNA1D, LCT, PRKCB, SLC37A4*	
